# One-tube detection of *Salmonella* Typhimurium using LAMP and CRISPR-Cas12b

**DOI:** 10.1128/spectrum.01271-24

**Published:** 2024-08-27

**Authors:** Jiansen Gong, Yi Jiang, Di Zhang, Tingting Li, Lixia Fu, Xinhong Dou

**Affiliations:** 1 Poultry Institute, Chinese Academy of Agricultural Sciences, Yangzhou, China; 2 Key Laboratory for Poultry Genetics and Breeding of Jiangsu Province, Jiangsu Institute of Poultry Sciences, Yangzhou, China; 3 College of Animal Science and Technology, Yangzhou University, Yangzhou, China; 4 Jiangsu Co-Innovation Center for the Prevention and Control of Important Animal Infectious Disease and Zoonose, Yangzhou University, Yangzhou, China; London Health Sciences Centre, London, Ontario, Canada

**Keywords:** *Salmonella *Typhimurium, LAMP, CRISPR/Cas12b, rapid detection, one-tube, one-step

## Abstract

*Salmonella enterica* serovar Typhimurium (ST) is a predominant serovar causing foodborne illnesses worldwide. Traditional detection methods often face challenges, including the need for specialized equipment, skilled operators, and lengthy procedures. To address these limitations, we developed a rapid, sensitive, and specific ST detection method by integrating loop-mediated isothermal amplification (LAMP) with the clustered regularly interspaced short palindromic repeats and associated protein 12b (CRISPR/Cas12b) system, all within a single tube. Our results indicate that the LAMP–CRISPR/Cas12b reaction can be completed isothermally in under 1 h without requiring specialized instruments. The platform’s limit of detection (LoD) is 12.5 copies per reaction. Additionally, the system demonstrated 100% inclusivity and exclusivity when tested against 30 reference strains, highlighting its specificity. In practical applications, the LoDs for ST in pure nucleic acid and contaminated fecal samples were 2.32 and 23.2 CFU/mL, respectively, with higher sensitivity observed in pure nucleic acid samples. Overall, our findings underscore the potential of the one-tube LAMP–CRISPR/Cas12b platform as a rapid, sensitive, and specific tool for ST detection, particularly in resource-limited settings.

**IMPORTANCE:**

Here, we have provided a novel one-step method for *Salmonella* Typhimurium detection in one pot by integrating the LAMP assay with the CRISPR/Cas12b system, offering significant advantages in terms of simplicity, speed, and accuracy.

## INTRODUCTION

*Salmonella enterica* serovar Typhimurium (ST) stands as a prevalent foodborne pathogen, widely distributed and transmitted through contaminated food globally (1). This serovar of *Salmonella* spp. is a significant contributor to salmonellosis in both humans and animals, characterized by its high fatality rates. Annually, ST accounts for more than 93.8 million cases of gastroenteritis and over 155,000 associated deaths, imposing substantial public health and socioeconomic burdens worldwide (2–4). In China, ST ranks among the top two dominant strains responsible for non-typhoidal *Salmonella* infections (5, 6). Consequently, advancing methods for early ST diagnosis is pivotal to mitigate food poisoning outbreaks and safeguard lives and assets.

While White–Kauffman serotyping has served as a primary *Salmonella* subtyping method for approximately 80 years, identifying over 2,600 *Salmonella* serovars (7), its time-consuming, imprecise, and low-sensitivity nature limits its efficacy. Although various rapid detection methods, including immunochromatographic strips, enzyme-linked immunosorbent assays, polymerase chain reaction (PCR), and loop-mediated isothermal amplification (LAMP), have been employed for ST detection (8–11), they often exhibit poor specificity, lengthy procedures, complex sample pretreatment, high false-positive rates, or elevated costs. Consequently, there remains a pressing need for a rapid, sensitive, and specific ST detection approach.

The clustered regularly interspaced short palindromic repeats and associated protein (CRISPR/Cas) system has emerged as a promising tool for bacterial detection due to the collateral cleavage activities of Cas effectors, such as Cas12a, Cas12b, and Cas13a (12). These effectors exhibit collateral cleavage activity on non-target single-stranded RNA and DNA following target sequence recognition and cleavage in the presence of specific RNA-guided nucleases. Several CRISPR/Cas-based platforms, including SHERLOCK, HOLMES, and DETECTR, have been utilized for nucleic acid analysis (13), with some specifically applied for ST detection (14–16). To enhance the sensitivity of traditional CRISPR-based methods and meet clinical pathogen testing demands, integrating the CRISPR/Cas system with isothermal amplification assays, such as LAMP or RPA, has been proposed. Ma et al. (17) developed a LAMP–CRISPR/Cas12a method for ST detection with remarkable sensitivity (14, 17). However, the separate amplification and detection steps in this approach risk contamination through multiple manual operations. Addressing this concern, Li et al. adapted HOLMES into the HOLMES v.2 platform for pathogen detection, quantifying target nucleic acids using a one-step system that combines LAMP amplification at a constant temperature, thus minimizing cross-contamination (15). Despite these advancements, a corresponding method tailored for ST detection remains elusive (16).

In this study, we integrate LAMP with clustered regularly interspaced short palindromic repeats and associated protein 12b (CRISPR/Cas12b) detection platform (LAMP–CRISPR/Cas12b) in a one-step, one-tube approach to develop a rapid, sensitive, and specific ST detection method. Our findings elucidate the optimal conditions for the one-step LAMP–CRISPR/Cas12b assay and underscore its potential advantages for identifying foodborne pathogens.

## RESULTS

### Schematic overview of the ST LAMP–CRISPR/Cas12b assay

**Fig 1:**
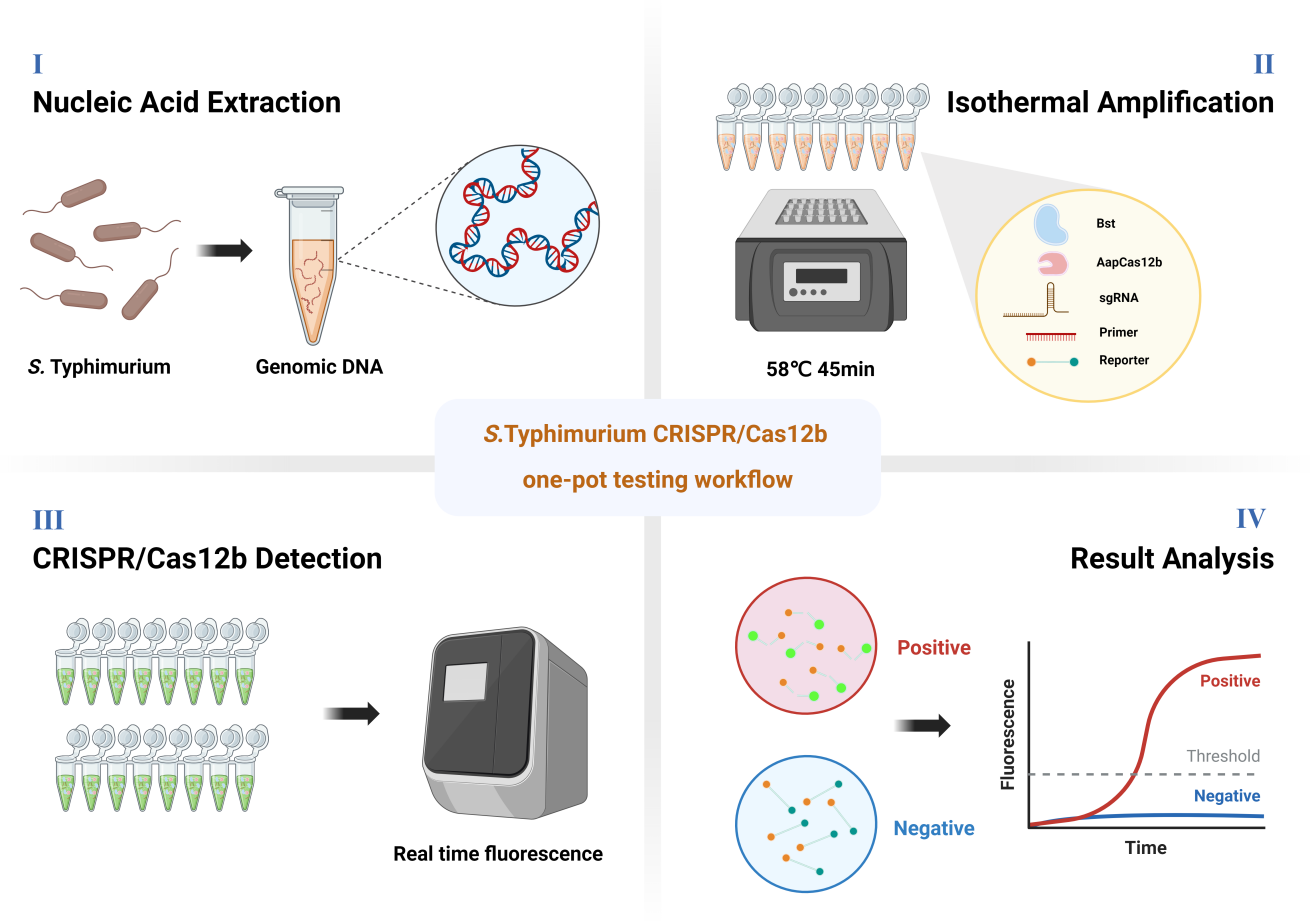
Illustration of the workflow for ST LAMP–CRISPR/Cas12b assay. The whole detection assay for CRISPR/Cas12b one-tube testing assay can be finished within 1 h at a constant temperature. The fluorescence signal can be detected in the presence of ST-positive samples. On the contrary, no significant fluorescence signal can be detected in the absence of ST-positive samples.

The workflow of ST LAMP–CRISPR/Cas12b assay is illustrated in Fig. 1. Briefly, the total DNA of ST was extracted by using a commercial DNA extraction kit. Then, DNA samples were firstly preamplified by LAMP reaction. Once the DNA amplicons were identified by the Cas12b crRNA, the trans-cleavage function could be activated and could cleave the single-strand DNA (ssDNA) fluorescence reporter (6-FAM/BHQ1 labeled), resulting in the appearance of fluorescence, then the results can then be detected on real-time PCR fluorescence readout instruments. Both the LAMP reaction and CRISPR/Cas12b detection were performed at a constant temperature of 58°C in one tube by one step. The whole procedure can be finished within 1 h, including 15 min for DNA extraction and 45 min for detection.

### Design and selection of LAMP primers for ST LAMP–CRISPR/Cas12b assay

**Fig 2:**
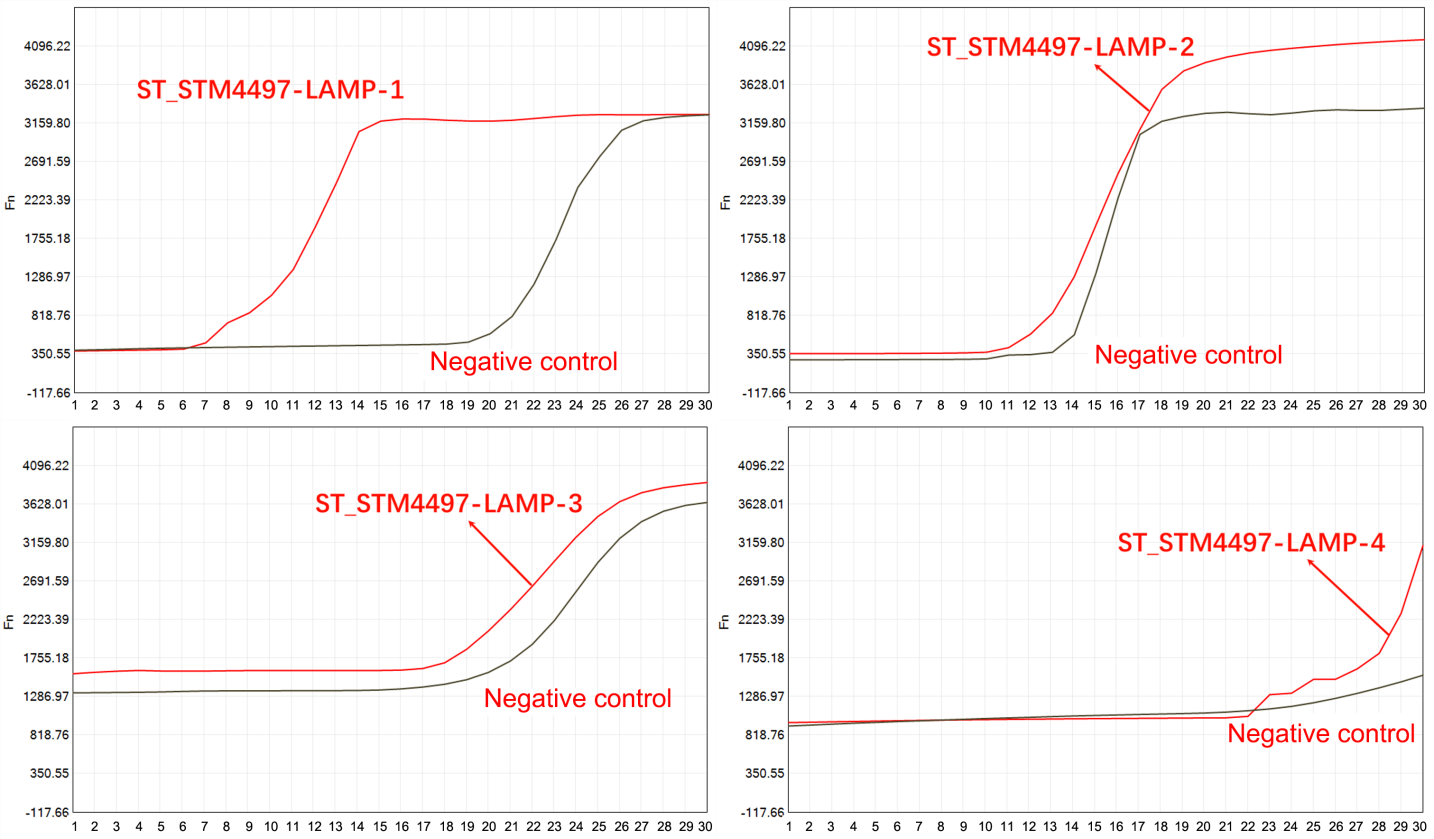
The amplification curve for ST target sequence by using four different LAMP primers as indicated. The horizontal axis displays the amplification time (minutes), and the vertical axis displays the fluorescence value. The figure shows that most of the primers functioned well, while ST_STM4497-LAMP-1 demonstrated the highest effectiveness. With ST_STM4497-LAMP-1, amplification started the earliest, and the negative control did not exhibit non-specific amplification.

*STM4497* (Gene ID: 1256023) was chosen as the target gene for ST in the present study, which is highly conserved and presents the specificity for ST (18, 19). Based on the sequence, a total of four pairs of LAMP primers named ST1–ST4 (Table 1) were designed via the Online NEB LAMP primer design tool (https://lamp.neb.com/#!/). All the primers were worked effectively to amplify the target gene. Moreover, the fluorescence take-off time of primer ST-1 is much earlier than that of their primers (Fig. 2). Therefore, primer ST-1 was chosen in the following reaction, where the sequences of LAMP primers were listed in.

**TABLE 1:** The LAMP primer used in the present study

LAMP primer		Sequence (5′−3′)
ST-1	F3-1	GATCGATCCCGTGCTTGAA
	B3-1	AGCATGTCGACGATGATCTG
	FIP-1	ACCGGAGCCGTTGTTTTTGAGATACCGCCTGTCACAGGTTC
	BIP-1	CTGCTCAGAATGAGCTGCAGGTCGCGAACTTGTGGTCCTT
	LF-1	AGCGCTCTTCGCTAATGCG
	LB-1	AACACCTGAAGTATCTGTTGCGT
ST-2	F3-2	CGTGCTTGAATACCGCCT
	B3-2	AGCATGTCGACGATGATCTG
	FIP-2	CCAATCTCATTACCGGAGCCGTACAGGTTCAGAGCCGCAT
	BIP-2	CTGCTCAGAATGAGCTGCAGGTCGCGAACTTGTGGTCCTT
	LB-2	GCGTGAACACCTGAAGTATCTGTTG
ST-3	F3-3	ACAGGAATGGGTAACGCCT
	B3-3	ACGAGGATTCAATGTCGCC
	FIP-3	GCAGGGGGGTAAAACCCATCACTGGTTCGTGGCCATGAG
	BIP-3	ATAGCGGACACGATCTCTCCCCTTCGGGTACCAGCCTGAA
	LB-3	GCGGGCATTGATTCCCGAAA
ST-4	F3-4	TTGAATACCGCCTGTCACAG
	B3-4	AGCATGTCGACGATGATCTG
	FIP-4	ACCCAATCTCATTACCGGAGCCTCAGAGCCGCATTAGCGA
	BIP-4	GCTCAGAATGAGCTGCAGGTGCCGCGAACTTGTGGTCCTT
	LB-4	GAACACCTGAAGTATCTGTTGCGT

### Synthesis of sgRNA for ST LAMP–CRISPR/Cas12b assay

According to the amplification product of primer ST-1, a specific ST sgRNA was synthesized and functioned well to recognize the target gene and activated the collateral activity of Cas12b, inducing the fluorescence appearance in the reaction. Therefore, the sgRNA as indicated above was used in the ST LAMP–CRISPR/Cas12b assay. The sequence of ST sgRNA is listed in Table 2.

**TABLE 2:** The sequence of ST sgRNA used in the present study^*a*^

ST sgRNA	Sequence (5′−3′)
sgRNA	GUCUAGAGGACAGAAUUUUUCAACGGGUGUGCCAAUGGCCACUUUCCAGGUGGCAAAGCCCGUUGAGCUUCUCAAAUCUGAGAAGUGGCAC**GGUUCUGGAUUUUUGAUUAU**

^*a*^ The target sequence is in bold font and underlined.

### Optimum conditions for ST LAMP–CRISPR/Cas12b assay

**Fig 3:**
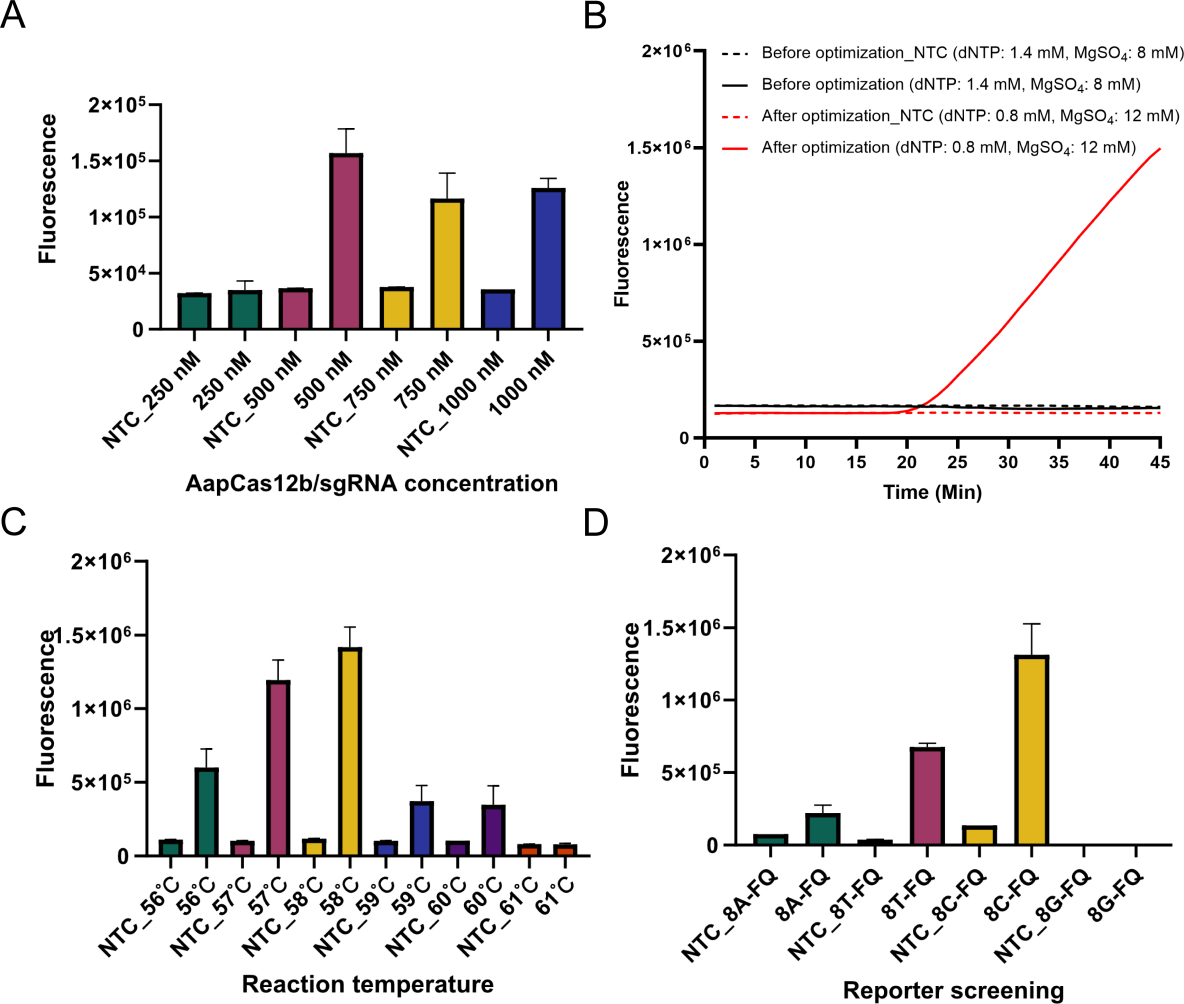
Determination of the optimum conditions for ST LAMP–CRISPR/Cas12b assay. (A) Cas12b and sgRNA (the testing group achieved the highest value in the concentration of 500 nM); (B) deoxy-ribonucleoside triphosphate (dNTP) and MgSO_4_ (the fluorescence intensity of test group was remarkably upregulated after optimization); (C) reaction temperature (the fluorescence intensity of the testing group achieved the highest value at 58°C); (D) ssDNA reporters (the 8C-FQ ssDNA reporter presented the best performance than that of other reporters for the reaction). Three replications were necessary for each reaction.

Firstly, the optimal concentrations of Cas12b protein and Cas12b sgRNA for the reaction were examined. Both the Cas12b protein and Cas12b sgRNA were diluted into four different concentrations and added to the reaction, respectively, including 250, 500, 750, and 1000 nM. As shown in Fig. 3A, the optimum reaction concentrations of Cas12b protein and Cas12b sgRNA were 500 nM, as it produced the highest fluorescence intensity.

Next, we examined the optimal dose of dNTP and MgSO_4_ for the assay. A set of concentration of dNTP (25 mM, 0.6–1.4 µL in 0.2-µL intervals) and MgSO_4_ (100 mM, 1.5–3.0 µL in 0.25-µL intervals) was adjusted and determined in the reaction. As shown in Fig. 3B, the combination of 0.8-µL dNTP and 3.0-µL MgSO_4_, corresponding to the working concentrations of 0.8 and 12.0 mM, respectively, showed the optimum detection results.

To confirm the optimal reaction temperature for the reaction, The reaction temperature was set from 56°C to 61°C with a 1°C increment. Clearly, the reaction condition in the temperature of 58°C showed the highest fluorescence intensity than that of other temperatures (Fig. 3C). Hence, 58°C was the optimum reaction temperature of the SE LAMP–CRISPR-top assay.

Moreover, a total of four HOLMES ssDNA reporters (named 8A-FQ, 8T-FQ, 8C-FQ, and 8G-FQ) were used in the present examination. The sequence information is listed in Table 3. Except for 8G-FQ, the other ssDNA reporters were able to produce fluorescence signals, especially for 8C-FQ. Therefore, the 8C-FQ reporter was applied in the ST LAMP–CRISPR-top assay (Fig. 3D).

**TABLE 3:** The HOLMES ssDNA reporters used in the present study

HOLMES ssDNA reporter	Sequence (5′−3′)
8A-FQ	5′-/6-FAM/AAAAAAAA/BHQ1/-3′
8T-FQ	5′-/6-FAM/TTTTTTTT/BHQ1/-3′
8C-FQ	5′-/6-FAM/CCCCCCCC/BHQ1/-3′
8G-FQ	5′-/6-FAM/GGGGGGGG/BHQ1/-3′

### Specificity and sensitivity of the ST one-step LAMP–CRISPR/Cas12b method

To examine the specificity of the present method, a set of DNA samples was extracted from 20 *Salmonella* spp. (including 3 ST strains and 17 other *Salmonella* strains) and 10 other foodborne bacteria. The detailed information is shown in Table 4.

**TABLE 4:** Strains used for testing the ST LAMP–CRISPR/Cas12b assay^*a*^

*Salmonella* strains	Source	Non-*Salmonella* strains	Source
*S*. Gallinarum	ATCC9184	*Proteus vulgaris*	CMCC49027
*S*. Blockley	CICC21489	*Serratia marcescens*	CMCC41002
*S*. Choleraesuis	CICC21493	*Escherichia coli*	ATCC25922
*S*. Agona	CICC21586	*Klebsiella pneumoniae*	CMCC46117
*S*. Thompson	CICC21481	*Shigella sonnei*	CMCC51592
*S*. Montevideo	CICC21588	*Shigella dysenteriae*	CMCC51105
*S*. Potsdam	CICC21500	*Enterococcus faecium*	ATCC35667
*S*. Kentucky	CICC21488	*Staphylococcus aureus*	ATCC25923
*S*. Heidelberg	CICC21487	*Bacillus cereus*	CMCC63303
*S*. Dublin	CMCC50042	*Campylobacter jejuni*	NCTC11168
*S*. Saintpaul	CICC21486		
*S*. Derby	CMCC50112		
*S. diarizonae*	ATCC12325		
*S. arizonae*	ATCC13314		
*S. bongori*	ATCC43975		
*S*. Indiana	ATCC51959		
*S*. Enteritidis	ATCC13076		
***S.* Typhimurium**	**ATCC14028**		
***S.* Typhimurium**	**CICC21485**		
***S.* Typhimurium**	**CMCC50115**		

^*a*^ The ST strains are in bold font and underlined.

**Fig 4:**
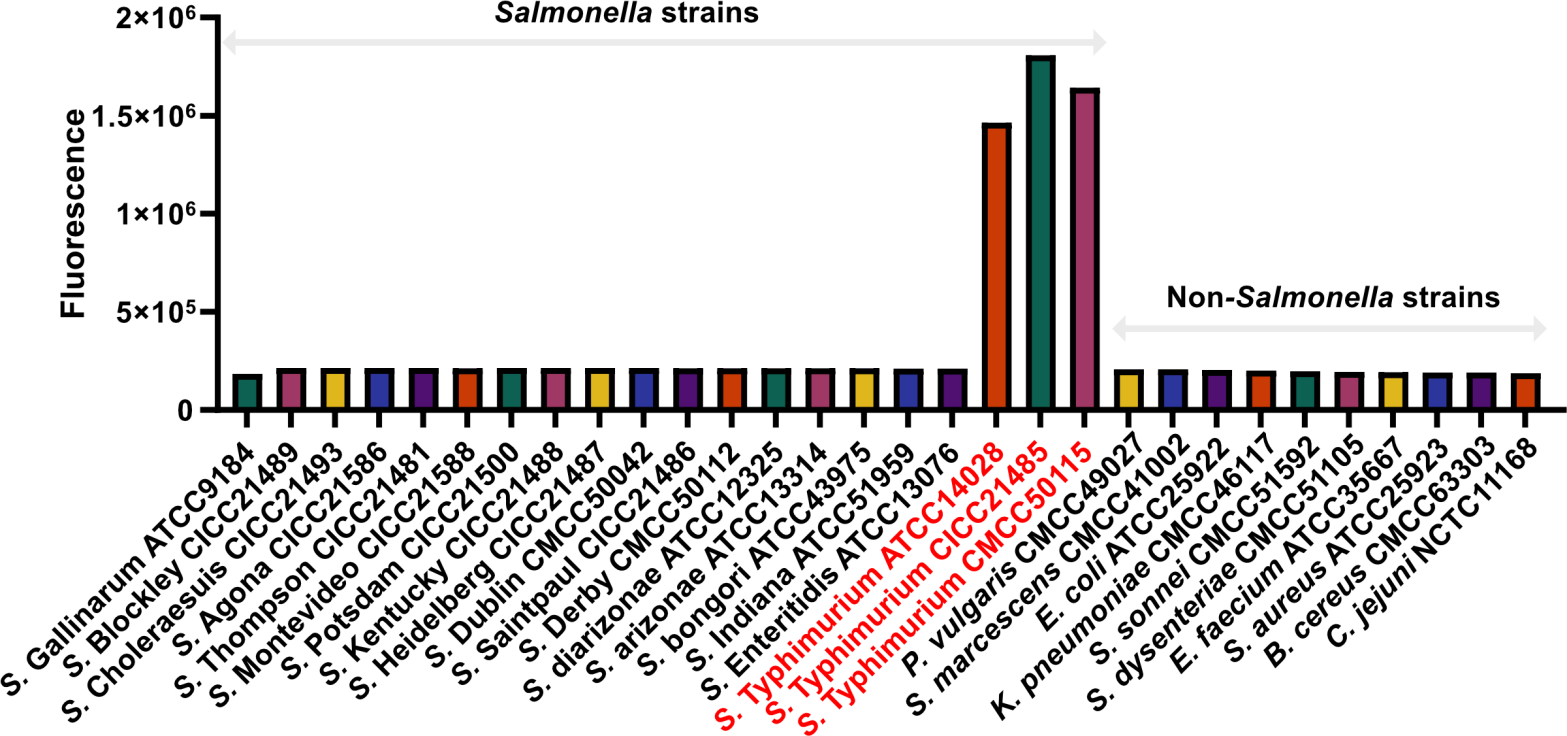
The specificity analysis of ST one-step LAMP–CRISPR/Cas12b. The three ST-positive samples were identified by using present LAMP–CRISPR/Cas12b reaction, while no cross-reaction was observed.

As shown in Fig. 4, all positive results were obtained from ST templates (3 of 3), whereas all negative results were obtained from non-ST templates (17 of 17) and other foodborne bacteria (10 of 10). Therefore, the specificity of the present method for ST is 100%, without non-specific false-positive results observed.

**Fig 5:**
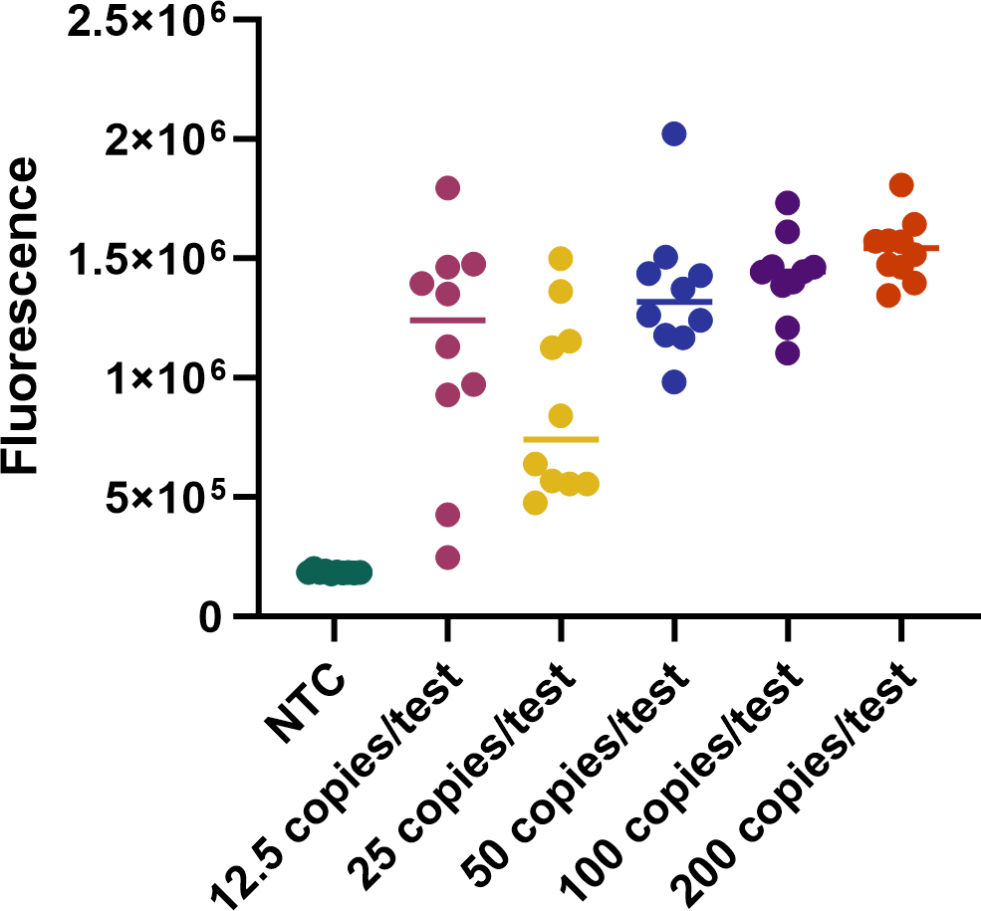
Determination of the LoD for ST one-step LAMP–ISPR/Cas12b assay. The LoD of ST one-step LAMP–CRISPR/Cas12b assay was achieved 12.5 copies per test. Ten replicates were performed for each reaction.

To analyze the limit of detection (LoD) of the ST one-step LAMP–CRISPR/Cas12b assay, the recombinant plasmid was diluted into 25, 50, 75, 100, and 125 copies per test. The sensitivity of the present assay was 12.5 copies per test (Fig. 5).

### Validation of the ST one-step LAMP–CRISPR/Cas12b method to ST contaminated chicken fecal samples

**Fig 6:**
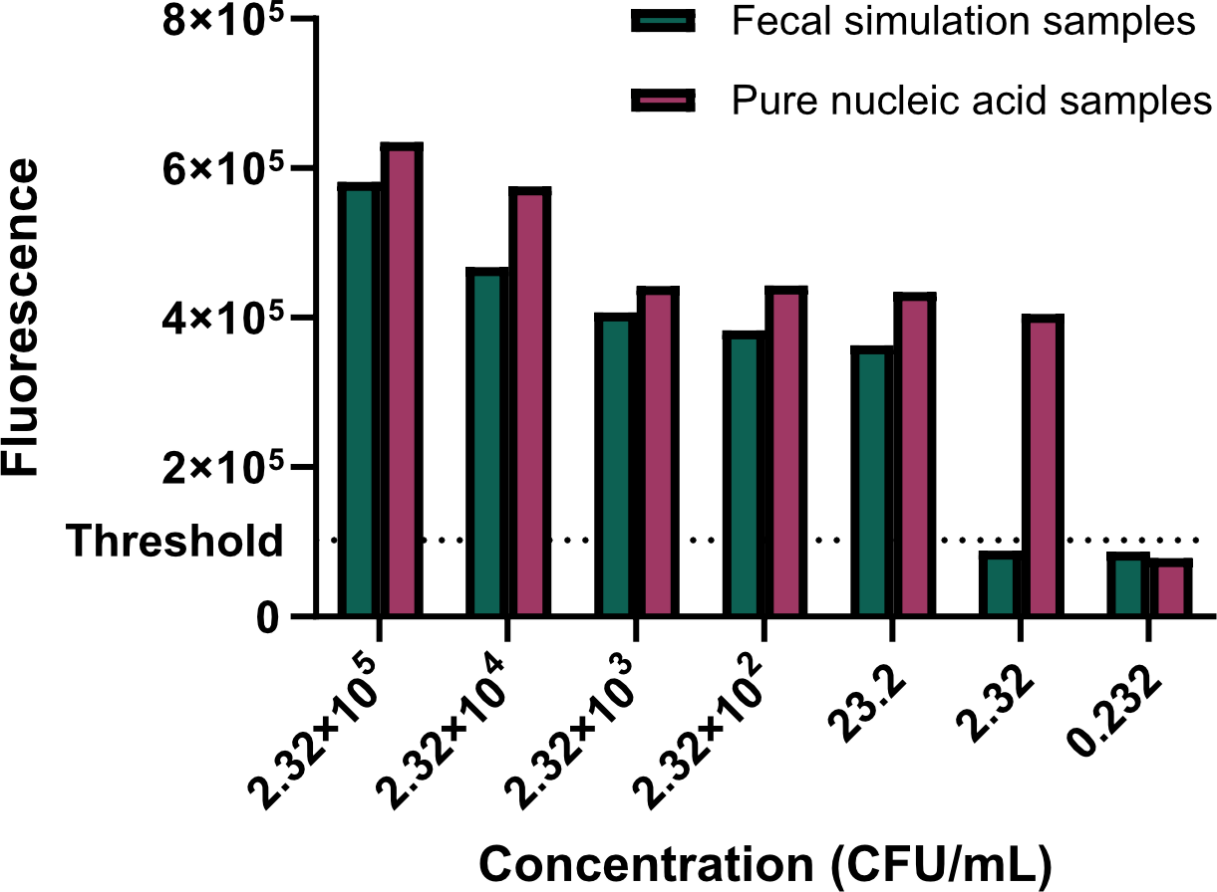
Practical application of LAMP–CRISPR/Cas12b method for ST-contaminated fecal. Both the ST-contaminated fecal and non-contaminated cultures were adjusted to the concentration from 10^−2^ to 10^5^ CFU/mL. The LoDs of the LAMP–CRISPR/Cas12b method for fecal simulation samples and pure nucleic acid samples were 23.2 and 2.32 CFU/mL, respectively.

To ascertain the practical application of LAMP–CRISPR/Cas12b method for ST, the genomic DNA samples of chicken fecal samples that were contaminated by using diluted ST cultures (10^−2^ to 10^5^ CFU/mL) were used for validation. The pure nucleic acid samples from non-fecal cultures were used as the positive control. As shown in Fig. 6, the present LAMP–CRISPR/Cas12 yielded the positive reaction in both contaminated fecal samples and pure ST nucleic acid samples in the dose from 10^1^ to 10^5^ CFU/mL, respectively. Moreover, the LoDs of this method for ST pure nucleic acid samples or contaminated fecal samples were 2.32 and 23.2 CFU/mL, respectively. Therefore, these data suggested that the LAMP–CRISPR/Cas12b method was more sensitive for detecting ST pure nucleic acid samples.

## DISCUSSION

ST ranks among the most severe foodborne pathogens, posing significant threats to public health and causing substantial economic losses (20). Alarmingly, the incidence of foodborne disease outbreaks is escalating annually (21). Despite advancements in foodborne pathogen detection methods, existing approaches often fall short of meeting the stringent demands of food safety monitoring. Consequently, there is an imperative need to innovate and refine methods for ST detection and identification.

While conventional culture-based methods for ST offer high sensitivity, they are labor-intensive and require over a week to yield results, making them impractical for high-volume testing (22, 23). PCR-based methods have gained popularity for pathogen detection due to their rapidity (24, 25). However, these methods are reliant on sophisticated equipment, skilled personnel, and extended reaction times, limiting their applicability in resource-limited settings. To address these challenges, isothermal amplification technologies, such as LAMP and RPA, have been introduced for nucleic acid detection. Notably, Edel et al. and Zhang et al. demonstrated the high sensitivity and specificity of LAMP-based methods for ST detection (26). Despite these advancements, the LAMP method’s susceptibility to false-positive results due to non-specific amplification remains a concern. To mitigate this issue, integrating the LAMP assay with the CRISPR/Cas detection system has been proposed for foodborne pathogen detection (27–29). However, its application for ST detection remains unexplored. In this study, we pioneered the integration of the LAMP assay with the CRISPR/Cas12b method, establishing a rapid, sensitive, and accurate one-tube, one-step reaction for ST identification.

Compared to traditional LAMP–CRISPR/Cas detection platforms, our approach eliminates the need for separate preamplification and target sequence detection steps. This integration minimizes cross-contamination risks associated with multiple manual operations, enhancing the method’s reliability.

The CRISPR/Cas12b system exhibits remarkable sensitivity to mismatches, with a single-nucleotide mutation in the target sequence’s first 18 nucleotides completely abolishing Cas12b cleavage activity (30, 31). This inherent specificity minimizes the risk of false-positive results. In our study, all positive ST samples were accurately identified without any cross-reactions, underscoring the system’s high specificity for ST detection.

Furthermore, the operational temperature range of Cas12b (37°C–60℃) overlaps with that of the LAMP assay (55°C–60℃) (32). Consequently, the LAMP assay can theoretically be seamlessly integrated with CRISPR/Cas12b in a one-tube, one-step format, eliminating the need for specialized equipment. Our findings corroborate this integration’s efficiency, demonstrating optimal performance at a constant temperature of 58℃, which can be achieved using compact and portable blue light instruments. Thus, our method offers a viable alternative for both laboratory and onsite ST detection.

The LoD is a crucial parameter for assessing detection method sensitivity. While PCR methods typically exhibit an LoD of 3 CFU/mL for ST detection (33), our LAMP–CRISPR/Cas12b platform achieved a comparable LoD of 2.32 CFU/mL with pure cultures, highlighting its superior sensitivity. Moreover, fecal samples serve as reliable indicators of systemic infection (34), and contaminated eggs correlate with fecal *Salmonella* levels. Our platform detected ST in contaminated fecal samples at a sensitivity of 23.2 CFU/mL, surpassing the performance of a colorimetric paper-based method, making it particularly well suited for ST spot identification (35).

In conclusion, we have developed a rapid, sensitive, and accurate ST detection method by integrating the LAMP assay with the CRISPR/Cas12b system in a one-tube, one-step format. Our findings not only furnish robust data supporting this innovative ST identification platform but also offer valuable insights for monitoring foodborne pathogens effectively.

## MATERIALS AND METHODS

### Reagents

The 10× LAMP buffer was purchased from Tolo Biotech (Shanghai, China); SYTO-9 was purchased from Thermo Fisher; AapCas12b was purchased from Tolo Biotech; HOLMES ssDNA reporter (FAM) was provided by Tolo Biotech; the Cas12b High Yield sgRNA Synthesis and Purification Kit was purchased from Tolo Biotech; the Digital PCR Mixture was purchased from Zhenzhun Bio (Shanghai, China). Nuclease-free water was purchased from Solarbio Life Sciences (Beijing, China).

### Bacterial culture and genomic DNA extraction

All bacterial strains except *Campylobacter jejuni* were cultured overnight at 37°C in brain heart infusion broth (Oxoid, Basingstoke, UK), while *Campylobacter jejuni* was cultured for 36 h at 42°C under microaerobic conditions in Mueller–Hinton broth (Oxoid). The bacterial genomic DNA extraction kit (Tiangen, Beijing, China) was used to extract the genomic DNA from the bacterial strains, in accordance with the manufacturer’s instructions. The extracted DNA was eluted in 100 µL of Tris-EDTA buffer and stored at −80°C until further analysis.

### LAMP primer and sgRNA design

The Online NEB LAMP primer design tool (https://lamp.neb.com/#!/) was used to design the LAMP primer set targeting the ST gene *STM4497* (Gene ID: 1256023). The Cas12b sgRNA was designed based on the coding sequence of *STM4497*. All the primers that used in the present study were synthesized in Sangon Biotech (Shanghai, China). The Cas12b sgRNA was purified by using Cas12b High Yield sgRNA Synthesis and Purification Kit (31904; ToloBio, China) according to the instruction of the manufacturer.

### LAMP reaction

The total volume of the LAMP reaction was 25 µL. The mixture includes 2.5 µL of 10× isothermal reaction buffer, 1.4-µL dNTP mix (25 mM), 1.75-µL MgSO_4_ (100 mM), 2.5-µL 10× ST *STM4497* LAMP primer mix, 6-µL glycine (2 M), 0.25-µL 100× SYTO-9, 1 µL of Bst 2.0 DNA polymerase, 2.5-µL template, and nuclease-free water up to 25 µL. LAMP ampliﬁcation was performed at 60°C for 45 min using pUC57-ST- *STM4497* plasmid and nuclease-free DW (NTC, negative control). The products of LAMP ampliﬁcation were monitored using the Applied Biosystems QuantStudio 5 Real-Time PCR System (QuantStudio 5; Thermo Fisher, USA). Three repeats were needed for each reaction.

### One-tube LAMP–Cas12b system

To identify the optimum condition for ST LAMP–CRISPR/Cas12b assay, the total volume of reaction mixture is 25 µL and prepared as follows: 2.5 µL 10× LAMP buffer, 0.8-/1.0-/1.2-/1.4-µL dNTP mix (25 mM), 1.5-/1.75-/2.0-/2.25-/2.5-µL MgSO_4_ (100 mM), 2.5-µL 10× ST *STM4497* LAMP primer mix, 6-µL glycine (2 M), 1.25-µL HOLMES ssDNA reporter (10 µM), 0.625-/1.25-/1.875-/2.5-µL AapCas12b (10 µM), 1-µL Bst (8 U/µL), 0.625-/1.25-/1.875-/2.5-µL S STM4497-sgRNA (10 µM), 2.5-µL template, and nuclease-free water up to 25 µL. The reaction was performed at 60°C for 45 min and monitored using a ﬂuorescence reader to collect the FAM fluorescent channel signals every 30 s.

### Fecal sample inoculation

The culture of ST strain ATCC14028 was incubated overnight at 37°C and reached the concentration of 2.32 × 10^9^ CFU/mL. Then, the culture was serially diluted 10-fold from 10^9^ to 10^−2^ by using nuclease-free water. The 1-g Specific pathogen Free (SPF) chicken feces was autoclaved and inoculated with 1 mL of diluted inoculum to obtain estimated inoculation levels of 10^−2^ to 10^5^ CFU/mL. Then, the genomic DNA samples were isolated from contaminated fecal cultures and used as the template for detection. The non-fecal cultures were used as the positive control.
